# Evaluation of Larvicidal Efficacy of *Ricinus communis* (Castor) Plant Extract and Synthesized Green Silver Nanoparticles against *Aedes albopictus*

**DOI:** 10.18502/jad.v14i2.3734

**Published:** 2020-06-30

**Authors:** Muhammad Waris, Shabab Nasir, Azhar Rasule, Iqra Yousaf

**Affiliations:** Department of Zoology, Government College University, Faisalabad, Pakistan

**Keywords:** Dengue mosquito, Larvicidal, *Ricinus communis*, Mosquito larvae, Silver nanoparticles (AgNPs)

## Abstract

**Background:**

*Aedes* mosquitoes are the most important group of vectors having ability of transmitting pathogens including arboviruses that can cause serious diseases like Chikungunya fever, Dengue fever and Zika virus in human. Biosynthesis and the use of green silver nanoparticles (AgNPs) is an important step in the search of reliable and ecofriendly control of these vectors.

**Methods:**

In this study an aqueous leaves extract of *Ricinus communis* (castor) and silver nanoparticles (AgNPs) synthesized from this extract were evaluated as larvicidal agent for 2^nd^ and 3^rd^ instar larvae of the *Aedes albopictus*. Different concentrations (50, 100, 150, 200 and 250ppm) of plant extract and synthesized nanoparticles were prepared and applied on second and third instar larvae. The percent mortality was noted after 6, 12, 18, 24, 30, 36, 42 and 48H of exposure and subjected to probit analysis to calculate LC_50_ and LC_90_.

**Results:**

Synthesized Ag^+^ nanoparticles were characterized by UV-Vis spectroscopy, Fourier transform infrared spectroscopy (FT-IR), and energy-dispersive X-ray spectroscopy (XRD). The nanoparticles were more toxic against larvae of *Ae. albopictus* with LC_50_ value (49.43ppm) and LC_90_ value (93.65ppm) for 2^nd^ instar larvae and LC_50_ (84.98ppm) and LC_90_ (163.89ppm) for 3^rd^ instar larvae as compared to the plant extract (149.58ppm, 268.93ppm) and (155.58ppm, 279.93ppm) for 2^nd^ and 3^rd^ instar larvae of *Ae. albopictus* respectively after 48H.

**Conclusion:**

Our results suggest the extract of *R. communis* and synthesized nanoparticles as excellent replacement of chemical pesticides to control the vector mosquitoes.

## Introduction

Mosquitoes cause a serious threat to public health (1–2). Vector-borne diseases such as malaria, dengue, chikungunya, Zika virus, Japanese encephalitis, filariasis, are being spread by mosquitoes (3). These diseases are found all over the world and cause millions of deaths annually (4). Pakistan is at the great risk of vector-borne diseases especially dengue due to it’s over crowded cities, insanitation and poor vaccination. In Pakistan, dengue cases are reported throughout the year but situation, usually, become worst in the post monsoon period (5). Pakistan had the worst dengue epidemic in 2011, during which more than 20,000 cases and 300 deaths were reported officially. In Khyber Pakhtunkhwa of Pakistan, July to end of September 2017 a total of, 52 926 cases of dengue fever including 38 deaths were reported (6) Chikungunya virus was detected in 1983 (7) and more than 4000 cases have confirmed through qualitative RT-PCR. Zika virus has reached near border areas in neighboring countries like China and India, so outbreak of the disease may occur in Pakistan (8).

The easy solution to avoid mosquito-borne diseases is the management of mosquito population. This management through chemicals causes health risks to human beings, environmental pollution and insecticidal resistance in mosquitoes (9). This prompted the need of searching for new chemical compounds and alternative strategies, as novel biological tools. So, medicinal plants can be used as an alternate for this purpose because these plants have many types of target specific, rapidly biodegradable, ecofriendly, and less toxic to human health larvicidal phytochemicals such as saponins, isoflavonoids, tannins, terpenes, steroids, etc. (10–11). Plants are good source of bioactive insecticidal phytochemicals that can kill mosquito larvae with high mortality rate (12–14) by bringing changes in development, midgut epithelium (15), mutation in DNA and production of reactive oxygen species (16–17). They have different mechanisms of action that reduce the chance of resistance development in mosquitoes (18).

**Fig. 1.**
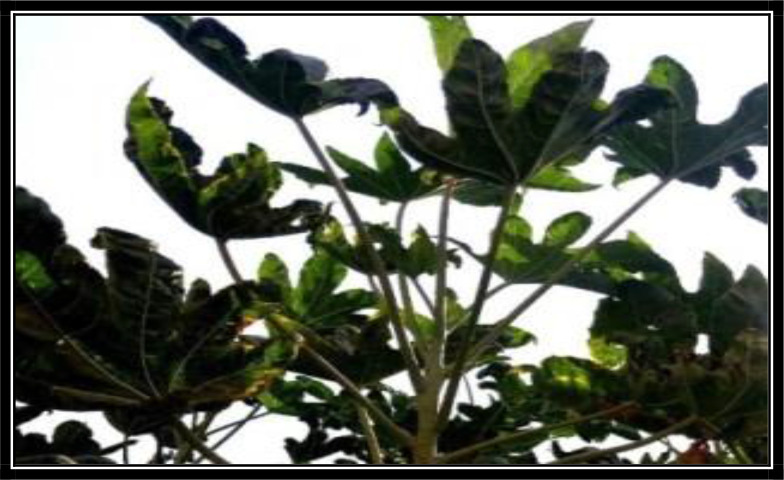
*Ricinus communis* (castor) plant (original photo)

Biologists have begun the use these phytochemicals as larvicides to control the mosquitoes (19). One step ahead, green silver nanoparticles (AgNPs) synthesized from plant extracts are proved more toxic than phytochemical as larvicides (20). Green silver nanoparticles (AgNPs) have high larvicidal effect because of small size ranging 1–100nm (21) and large surface area. These characteristics of AgNPs made them a unique larvicide at very low concentrations. These have been tested in a variety of entomological research because these are safe, low cost, easily available and have a simple easy biosynthesis process (21–22). *Ricinus communis* (castor) plant belongs to a big family Euphorbiaceae contains nearly about 300 genera and 7500 species. *Ricinus communis* (castor) is a flowering plant, has high medicinal value for healthy human life. This plant is used as laxative, fungicide, anti-oxidant, anti-asthmatic, antiulcer, wound healing, insecticidal and larvicidal agent. It has important phytochemicals like glycosides, alkaloids, flavonoids, steroids and saponins. that are helpful in controlling mosquitoes (23–24) (Fig. 1). Due to these reasons, the present study was designed to evaluate the larvicidal potential of the plant extract and AgNPs synthesized from this extract of *R. communis* (castor) against 2^nd^ and 3^rd^ larvae of *Ae. albopictus* under laboratory conditions. UV-Vis spectroscopy analysis, Powdered X-ray diffraction (PXRD) and Fourier Transform Infrared Radiation (FTIR) spectroscopy were used to confirm the biosynthesis of AgNPs.

## Materials and Methods

### Preparation of leaf extract

Healthy and fresh leaves of the *R. communis* (castor) plants were collected (hand plucked) from the old campus of University of Agriculture Faisalabad, Pakistan during the month of May, 2017. Leaves were cleaned with cloth and washed with tap water to remove dust. Then the leaves were dried in shady place at room temperature and grinded in an electric grinder (Anex Germany) (25). Fifty grams powder of leaves was mixed with 250ml acetone as solvent in the Soxhlet apparatus and boiled gently at boiling point range 55.5–56.5 °C for complete extraction (8h) and stored at 4 °C (26).

### Preparation of Green AgNPs

Silver nitrate (AgNO_3_) of Sigma was purchased and 1mM solution of silver nitrate (AgNO_3_) was prepared in 250mL Erlenmeyer flask in the darkness to avoid action of light. 10ml acetone plant extracts of *R. communis* (castor) was put in 250ml conical flask having 90ml of 1mM silver nitrate solution. Two to three drops of 1% NaOH were added for the adjustment of pH at 8 and mixed continuously by magnetic stirrer. This mixture was kept at 40 °C for one hour under clear sky condition for irradiation. Colour change of the solution indicated the formation of AgNPs. Reaction completed on attaining reddish brown colour. Solution was cooled and stored in amber bottle at 4 °C, then centrifuged for three times at 5000rpm for 20 minutes to obtain pellets. Purified suspension was made by dissolving pellets in double distilled water and was frozen for further use (27).

### Characterization of AgNPs.

The biosynthesized silver nanoparticles were characterized by UV-Vis spectroscopy analysis, Powdered X-ray diffraction (PXRD) and Fourier Transform Infrared Radiation (FTIR) spectroscopy assistance through High Tech central laboratory of Government College University Faisalabad.

### UV-Vis absorbance spectroscopy.

To monitor formation of the green silver nanoparticles, absorption spectra were taken at a scanning speed of 200 to 800nm using a Cary 60 double beam UV-Vis spectrophotometer (Spectramax M3 molecular devices) operating at the resolution of 1nm. UV-Vis spectra were recorded after 15 and 30 minutes (28).

### Powdered X-ray Diffraction (PXRD)

The shape of structure and size of the silver nanoparticles was calculated through diffracted intensities at 40kV voltage and 30mA current with the range of 0°–80° 2θ in CuKα radiation (Rigaku, Ultima IV, and X-ray diffractometer system).

### Fourier Transform Infrared (FTIR) Spectroscopy

The residue solution of 100ml was centrifuged at 5,000rpm for 10 minutes after reaction to remove impurities. To obtain pure pellet of AgNPs the supernatant was again centrifuged at 10000rpm for 60 minutes. All measurements were carried out in the range of 400–4000cm^−1^ at a resolution of 4cm^−1^ (29). Fresh samples having volume of 1–2ml in aqueous form were sent for FTIR Analysis to Hi-Tech Lab, Government College University Faisalabad.

(1) $$\text{Percentage}\;\text{Mortality}\;=\;\frac{\text{Number}\;\text{of}\;\text{dead}\;\text{larvae}}{\text{Number}\;\text{of}\;\text{larvae}\;\text{introduced}}\times 100$$

### Collection and rearing of mosquitoes

Larvae and pupae were collected with dippers from a forest near Faisalabad, Punjab, Pakistan (31° 25′ 7.3740″ N and 73° 4′ 44.7924″ E, 192 meters above the sea level). Collected immature stages of mosquito were brought back to the Zoology Lab, department of Zoology, Government College University, Faisalabad, inside beakers closed with muslin cloth. Larvae and pupae of *Ae. albopictus* were identified with the help of identification keys (30–31), reared to adults in 1000ml beakers containing water under lab conditions at 25±5 °C and 80±5% RH (32). Adults further reared in separate glass cages. Male adults were fed with 10% sugar solution and females with blood on live white rats in separate glass cages for egg laying (33). Larvae emerged from the eggs were reared in batches of 300 each, in 1200ml deionized water in stainless steel trays (35x 30x 5cm) for the bioassays (34). Fifth generation larvae were used for the bioassay.

### Bioassay

Group of 20 actively swimming 2^nd^ and 3^rd^ instar larvae (identified from the shed cuticle and from the size and colour of the larvae) of *Ae. albopictus* were released in 250ml beaker containing 200ml distilled water separately. Five concentrations including 50, 100, 150, 200 and 250ppm of larvicidal solution of *R. communis* extract and green AgNPs synthesized from the extract were prepared using distilled water and subjected for mortality assays separately. Bioassay was conducted at 27±3 °C, 80±3% relative humidity (RH) and a photoperiod of 16: 8 (L: D) hours (35). The control was set up with dechlorinated tap water and five replications were done for each treatment. Mortality rates were calculated using the WHO (3) bioassay protocol with slight modifications. The percentage mortalities were corrected by using Abbott’s formula (36).

(2) $$\text{Corrected}\;\text{mortality}\;=\;\frac{\text{Observed}\;\text{mortality}\;\text{in}\;\text{treatment}-\text{Observed}\;\text{mortality}\;\text{in}\;\text{control}}{100-\;\text{Control}\;\text{mortality}}\times 100$$

The average larval mortality data was subjected to probit analysis using Minitab −17 statistical software (2017) for calculating lethal concentration 50% (LC_50_) and 90% (LC_90_) of larvae and for getting dose and time mortality regression lines.

## Results

### Synthesis of Silver Nanoparticles

**Fig. 2.**
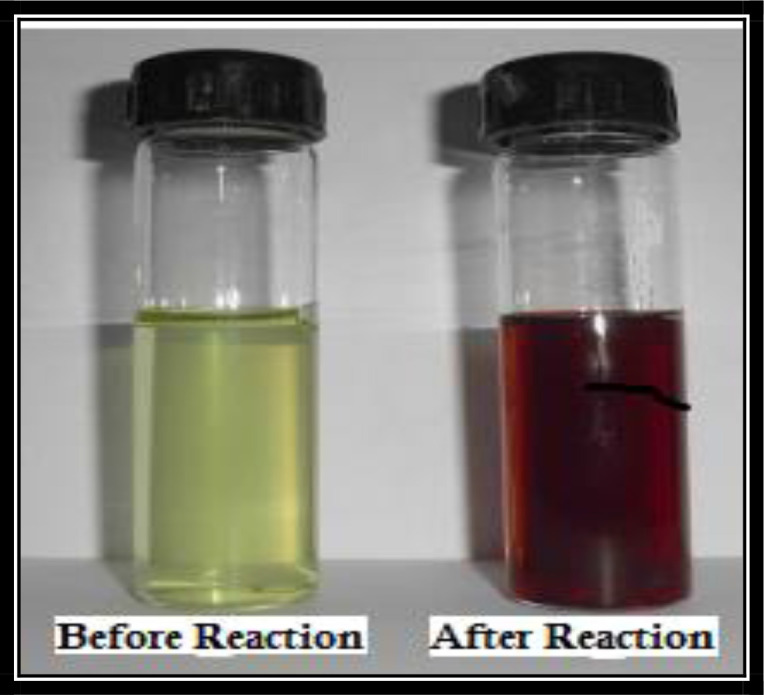
Bioreduction of AgNO_3_ into AgNPs using plant extract (colour change)

Formation of green AgNPs through the reduction of the silver metal ions by the extract of *R. communis* that turned the colour of mixture (plant extract +AgNO_3_ Solution) into reddish brown in 1H at 40 °C (Fig. 2).

### UV-Vis spectrum of silver nanoparticles

**Fig. 3.**
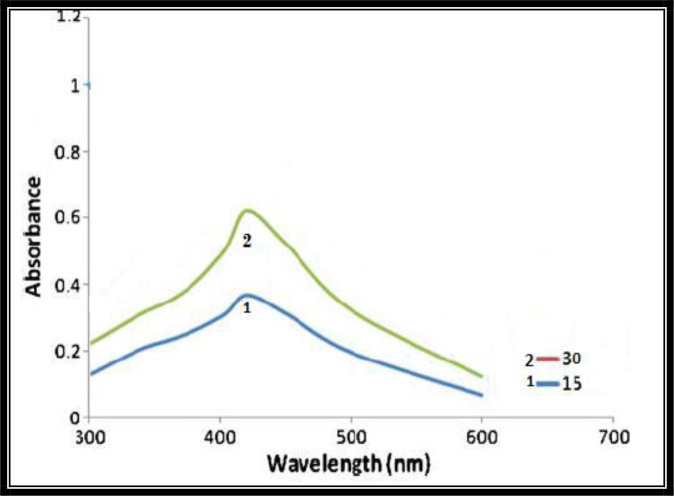
Ultraviolet-Visible spectra of silver nanoparticles synthesized by treating *R. communis* leaf extract with 1 mM AgNO_3_ solution

The progress of the reaction between Ag^+^ and the leaf extract was monitored by UV–visible spectra of silver nanoparticles in aqueous solution with different reaction times that are shown (Fig. 3). It was observed from the figure that localized surface plasmon resonance band showed maximum absorbance at 430nm after 30 minutes of reaction time.

### Powdered X-ray Diffraction (PXRD) Studies

**Fig. 4.**
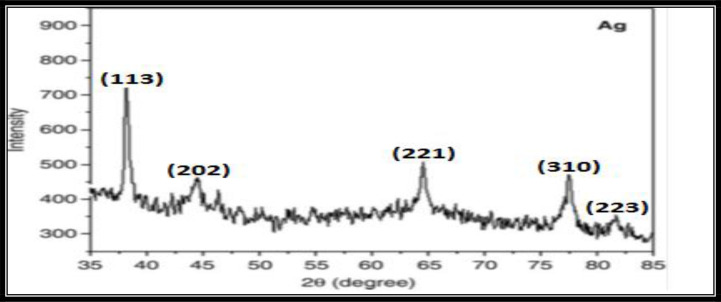
Powdered X-ray diffraction

Result of PXRD (Fig. 4) showed intense silver nanoparticle (AgNPs) diffraction peaks at 38.10, 44.46, 64.45, 77.51, and 81.60, corresponding to facets 113, 202, 221, 310, and 223 of the face-centered cubic crystal structure.

### Fourier Transform Infrared Radiation Spectroscopy (FTIR) Analysis

**Fig. 5.**
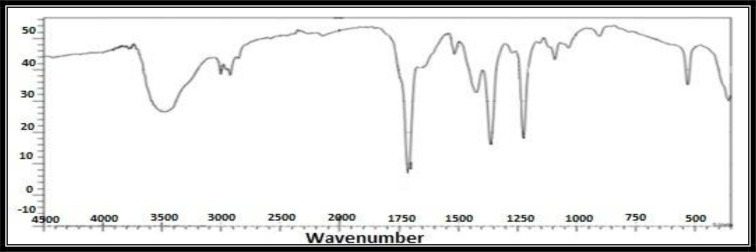
Fourier Transform Infrared Radiation spectra of AgNPs synthesized from leaf extract of *R. communis* (Castor)

The FTIR spectra of silver nanoparticles prepared from the *R. communis* leaf extract (Fig. 5) showed transmittance peaks at 1263.2, 978.6, 849.1, 710.5, 662.8, 502.7, and 435.6/cm. The carbonyl group formed amino acid residues which capped the silver nanoparticles indicated by these peaks. These residues prevent from agglomeration of AgNPs, and made the medium stable. FTIR clearly indicate role of proteins and other compounds of leaf extract in the formation and stabilization of AgNPs.

### Larvicidal activity of leaf extracts and synthesized silver nanoparticles

The results of the larvicidal activity of leaf extract of *R. communis* (Castor) and synthesized AgNPs with different concentrations (50–250ppm) after different exposure times (6, 12, 18, 24, 30, 36, 42 and 48H) showed a dose and time-dependent toxic effects against 2^nd^ and 3^rd^ instar larvae of *Ae. albopictus*. No mortality was observed in the control group. AgNPs synthesized from *R. communis* showed 100% mortality for all the exposed larvae after 36H at the concentration of 250ppm (Table 1). The synthesized AgNPs showed least values of LC_50_ (49.43ppm) and LC_90_ (93.65ppm) after 48H with regression equation Y= −1.208+0.1521x against 2^nd^ instar larvae of *Ae. albopictus*. Similarly for the 3^rd^ instar larvae, the least values of LC_50_ and LC_90_ were 84.98 and 163.89ppm respectively after 48H with regression equation Y= −1.072+0.1461x as shown in Table 1.

**Table 1.** Larvicidal activity of AgNPs synthesized from *R.communis* against *Ae. albopictus* larvae

**Time**	**Larval instars**	**% Mortality±SD**	**Lethal concentration**		**FL at 95% C.I**	**Chi-square**	**P value**	**Regression equation**
**6H**	2^nd^	10±0.27	LC_50_	523.02	384.98–1129.65	3.52	0.319	Y= −2.30+0.0044x
			LC_90_	813.34	564.21–1925.07	3.52	0.319	Y= −2.30+0.0044x
	3^rd^	9±0.27	LC_50_	565.14	400.52–1494.50	3.72	0.292	Y= −2.27+0.0040x
			LC_90_	883.53	589.09–2568.35	3.72	0.292	Y= −2.27+0.0040x
**12H**	2^nd^	15±0.24	LC_50_	471.43	363.43–827.90	2.16	0.539	Y= −2.19+0.0046x
			LC_90_	747.16	543.88–1430.95	2.16	0.539	Y= −2.19+0.0046x
	3^rd^	10±0.21	LC_50_	513.08	380.19–1034.82	1.34	0.712	Y= −.96+0.0038x
			LC_90_	847.98	591.32–1873.27	1.34	0.712	Y= −1.96+0.0038x
**18H**	2^nd^	20±0.20	LC_50_	437.37	344.45–706.71	0.54	0.909	Y= −1.96+0.0038x
			LC_90_	728.90	541.17–1285.97	0.54	0.909	Y= −1.96+0.0038x
	3^rd^	16±0.21	LC_50_	513.08	380.19–1034.82	0.13	0.718	Y= −.96+0.0038x
			LC_90_	847.98	591.32–1873.27	0.13	0.718	Y= −1.96+0.0038x
**24H**	2^nd^	25±0.19	LC_50_	384.70	316.42–547.57	0.84	0.809	Y= −1.89+0.0049x
			LC_90_	645.16	501.53–998.63	0.84	0.809	Y= −1.96+0.0038x
	3^rd^	20±0.19	LC_50_	455.96	351.97–786.41	0.17	0.918	Y= −1.83+0.0040x
			LC_90_	774.01	561.84–1463.36	0.17	0.918	Y= −1.83+0.0040x
**30H**	2^nd^	50±0.16	LC_50_	245.24	222.17–280.11	0.34	0.809	Y= −1.70+0.0059x
			LC_90_	430.12	526.76–372.84	0.34	0.809	Y= −1.70+0.0059x
	3^rd^	35±0.19	LC_50_	339.95	291.79–435.60	0.17	0.244	Y= −1.99+0.0058x
			LC_90_	558.36	455.79–770.54	0.17	0.244	Y= −1.99+0.0058x
**36H**	2^nd^	70±0.15	LC_50_	188.19	172.39–207.37	0.77	0.809	Y= −1.42+0.0075x
			LC_90_	357.11	317.50–418.58	0.77	0.809	Y= −1.42+0.0075x
	3^rd^	50±0.16	LC_50_	242.61	220.36–275.70	4.17	0.253	Y= −1.719+0.0070x
			LC_90_	423.45	368.57–514.79	4.17	0.253	Y= −1.719+0.0070x
**42H**	2^nd^	100±0.15	LC_50_	104.99	90.40–117.45	5.77	0.033	Y= −1.43+0.1545x
			LC_90_	217.85	202.21–238.34	5.77	0.033	Y= −1.43+0.1545x
	3^rd^	95±0.14	LC_50_	115.02	103.75–125.31	1.67	0.000	Y= −1.077+0.0108x
			LC_90_	229.82	211.09–225.49	1.67	0.00	Y= −1.077+0.0108x
**48H**	2^nd^	100±0.12	LC_50_	49.43	37.51–59.44	6.77	0.032	Y= −1.208+0.1521x
			LC_90_	93.65	81.06–110.33	6.77	0.032	Y= −1.208+0.1521x
	3^rd^	100±0.12	LC_50_	84.98	70.40–101.45	1.27	0.303	Y= −1.077+0.1461x
			LC_90_	163.89	151.09–175.49	1.27	0.303	Y= −1.077+0.1461x

LC_50_: lethal concentration that kills 50% of the exposed larvae; LC_90_: lethal concentration that kills 90% of the exposed larvae.

The mortality rate of 2^nd^ and 3^rd^ instar larvae of *Ae. albopictus* was noted as 98 and 96% respectively after 48H at 250ppm concentration of *R. communis* leaves extract (Table 2). The least values of LC_50_ and LC_90_ were 149.57 and 268.92ppm for 2^nd^ instar larvae and 155.57 and 279.92ppm for 3^rd^ instar respectively with regression equations Y= −1.16+0.129x and Y= −1.210+0.113x after 48H. The extract of *R. communis* exhibited prominent larvicidal activity against the 2^nd^ instar larvae of *Ae. albopictus*. All the concentrations of plant extracts used in the present study exhibited repellency activity.

**Table 2.** Larvicidal activity of leaf extracts of *R. communis* against *Ae.s albopictus* larvae

**Time**	**Larval instars**	**%Mortality±SD**	**Lethal concentration**		**FL at 95% C.I**	**Chi-square**	**P value**	**Regression equation**
**6H**	2^nd^	9±0.28	LC_50_	572.94	403.52–1584.71	5.12	0.163	Y= −2.31+0.004x
			LC_90_	889.79	589.68–2705.85	5.12	0.163	Y= −2.31+0.004x
	3^rd^	6±0.27	LC_50_	725.56	448.49–1927.47	4.22	0.238	Y= −2.19+0.003x
			LC_90_	1148.36	664.68–2035.2	4.22	0.238	Y= −2.19+0.003x
**12H**	2^nd^	15**±**0.22	LC_50_	537.82	403.69–1002.15	1.35	0.717	Y= −2.06+0.041x
			LC_90_	778.85	559.72–1546.13	1.35	0.717	Y= −2.06+0.041x
	3^rd^	11**±**0.27	LC_50_	725.56	448.49–1927.47	4.22	0.238	Y= −2.19+0.003x
			LC_90_	1148.31	664.68–2035.20	4.22	0.238	Y= −2.19+0.003x
**18H**	2^nd^	25**±**0.20	LC_50_	407.93	329.78–608.36	0.92	0.81	Y= −2.06+0.041x
			LC_90_	679.10	518.69–1101.86	0.92	0.81	Y= −2.06+0.041x
	3^rd^	18**±**0.19	LC_50_	507.42	374.13–1045.96	0.06	0.995	Y= −1.79+0.003x
			LC_90_	869.61	601.13–1974.48	0.06	0.995	Y= −1.79+0.003x
**24H**	2^nd^	30**±**0.16	LC_50_	313.16	265.99–411.52	2.08	0.554	Y= −1.48+0.005x
			LC_90_	583.50	465.24–845.33	2.08	0.554	Y= −2.06+0.041x
	3^rd^	22**±**0.19	LC_50_	470.34	356.20–869.28	0.49	0.921	Y= −1.18+0.004x
			LC_90_	820.08	582.04–1670.4	0.49	0.921	Y= −1.18+0.004x
**30H**	2^nd^	40**±**0.16	LC_50_	313.16	265.99–411.52	2.08	0.554	Y= −1.48+0.005x
			LC_90_	583.50	465.24–845.33	2.08	0.554	Y= −1.48+0.041x
	3^rd^	30**±**0.18	LC_50_	370.21	303.81–530.08	0.054	0.997	Y= −1.65+0.004x
			LC_90_	656.65	506.69–1031.3	0.054	0.997	Y= −1.65+0.004x
**36H**	2^nd^	50**±**0.16	LC_50_	258.97	229.21–309.36	0.67	0.880	Y= −1.46+0.006x
			LC_90_	485.52	406.70–633.31	0.67	0.880	Y= −1.46+0.046x
	3^rd^	40**±**0.17	LC_50_	317.15	272.97–403.39	2.05	0.532	Y= −1.71+0.005x
			LC_90_	554.63	451.92–766.05	2.05	0.532	Y= −1.71+0.005x
**42H**	2^nd^	88**±**0.15	LC_50_	197.93	176.04–209.75	3.26	0.114	Y= −1.51+0.153x
			LC_90_	323.98	301.79–336.20	3.26	0.114	Y= −1.51+0.153x
	3^rd^	87**±**0.15	LC_50_	276.36	244.35–288.73	0.55	0.832	Y= −1.517+0.010x
			LC_90_	372.02	350.53–401.52	0.55	0.832	Y= −1.517+0.010x
**48H**	2^nd^	98**±**0.14	LC_50_	149.57	138.29–159.74	2.06	0.518	Y= −1.16+0.129x
			LC_90_	268.92	254.26–280.00	2.06	0.518	Y= −1.16+0.129x
	3^rd^	96**±**0.15	LC_50_	155.57	139.29–170.74	1.65	0.102	Y= −1.210+0.113x
			LC_90_	279.92	252.26–295.00	1.65	0.102	Y= −1.210+0.113x

LC_50_: lethal concentration that kills 50% of the exposed larvae; LC_90_: lethal concentration that kills 90% of the exposed larvae

## Discussion

Nanotechnology is an emerging technology in modern era that enables scientists to synthesize particles of different sizes, forms and components. Hence synthesized nanoparticles of gold, silver and platinum are being used for insect vectors control and in pharmaceutical industries (17). During current study, change in colour (reddish brown) of the solution indicated the formation of AgNPs due to the reduction of silver metal ions by the extract of *R. communis* and was confirmed by the localized surface plasmon resonance band absorbance at 430nm after 30 minutes of reaction time. Previous studies also indicated the similar colour changes and maximum absorbance at 430nm (37). Our result of PXRD indicated intense silver nanoparticle (AgNPs) diffraction peaks at 38.10, 44.46, 64.45, 77.51 and 81.60 corresponding to facets 113, 202, 221, 310 and 223 of the face-centered cubic crystal structure. Sathyavathi et al. (25) also reported diffraction peaks at 44.50, 52.20, and 76.7, which correspond to the 111, 200, and 222 facets of the face-centered cubic crystal structure. XRD result of silver nanoparticles reported by Nirmala et al. (38) is also close to the cited results.

The FTIR spectra of current study showed transmittance peaks at 1263.2, 978.6, 849.1, 710.5, 662.8, 502.7, and 435.6/cm. The carbonyl group formed amino acid residues which capped the silver nanoparticles indicated by these peaks. These residues prevent from agglomeration of AgNPs, and made the medium stable. FTIR clearly indicate role of proteins and other compounds of leaf extract in the formation and stabilization of AgNPs (37–40).

In our study, the *R. communis* AgNPs showed 100% mortality at 250ppm for *Ae. albopictus* after 48H, with LC_50_ and LC_90_ values 49.43, 93.65ppm and 84.98, 163.89ppm for 2^nd^ and 3^rd^ instar larvae respectively, while LC_50_ and LC_90_ values of the leaf extract of *R*. *communis* after 48h exposure were 149.57, 268.92ppm and 155.57, 279.92ppm for 2^nd^ and 3^rd^ instar larvae respectively. These results clearly indicated that *R. communis* AgNPs were more potent than leaf extract of *R*. *communis* due to less LC_50_ and LC_90_ values. These results are in line with the results of other scientists who also reported more potency of AgNPs than simple plant extracts (38–39).

Karthikeyan et al. (39) also reported the toxicity of AgNPs synthesized from *Euphorbia hirta* leaf extract against *An.stephensi* 1^st^ to 4^th^ instar larvae with LC_50_ (10.14, 16.82, 21.51, and 27.89ppm, respectively) after 24H. This high potency of AgNPs was due to high surface area-to-volume ratio, that imparts different biological and catalytic activities in them (30–40). Hemant et al. (40) also reported the potency of AgNPs of *Euphorbia tirucalli* against 2^nd^ and 4^th^ instar larvae with least LC_50_ values (3.50 to 7.01ppm) and (4.44 to 8.74ppm) respectively after 24 hrs.

Our findings are at par with the previous findings that simple leaf extracts are less potent than AgNPs. The larvicidal effects of leaves extracts of *R. communis* showed the LC_50_ values of 1091.44, 1364.58 and 1445.44ppm against 2^nd^, 3^rd^ and 4^th^ larval instars of *Cx. quinquefasciatus* (41). Basheer (42) also used *R. communis* extracts through different solvents and found ethyl acetate extract more potent than others against 3^rd^ instar larvae of *Ae. albopictus*. He also noted that LC_50_ values decreased with time. These results are similar with our findings. Mandal (43) also noted that *R. communis* seed extract exhibited larvicidal effects with 100% mortality at concentrations 32–64μg/mL, with LC_50_ value 16.84μg/mL for *Ae. albopictus* larvae. All previous scientists studied either plant extracts or their AgNPs separately but the present study compared the plant extract with its AgNPs. Cited results are close to our findings but not exactly same due to using different plant, mosquito species, larval stage and solvent for plant extraction.

Results of our study suggested that the leaf extract of *R. communis* is toxic to *Ae. albopictus* larvae and toxicity increased when extract combined with AgNPs. Our results clearly proved the excellent larvicidal efficacy of *R. communis* against *Ae. albopictus*.

## Conclusion

It is concluded from our findings that, the leaf extract and synthesized silver nanoparticles of *R. communis* had excellent potential for killing the of mosquito larvae. The application of this plant extract along with silver nanoparticle on mosquito breeding places surely decrease the population of vector mosquitoes, control many dreadful diseases and prevent environmental pollution.
